# Isolated Posterior Horn Medial Meniscus Bony Avulsion: A Case Report

**DOI:** 10.1155/cro/7859802

**Published:** 2026-09-02

**Authors:** Ahmad R. Alhankawi, M. Lane Moore, Don L. Dulle, Matthew B. Anastasi, Anikar Chhabra

**Affiliations:** ^1^ Mayo Clinic Alix School of Medicine, Mayo Clinic, Scottsdale, Arizona, USA, mayoclinic.org; ^2^ Department of Orthopedic Surgery and Sports Medicine, Mayo Clinic, Phoenix, Arizona, USA, mayoclinic.org

**Keywords:** arthroscopy, knee, meniscus, root avulsion

## Abstract

**Background:**

The menisci provide mechanical stability and shock absorption in the knee joint, distributing stress across the tibial plateau. Root tears to the menisci are functionally equivalent to a total meniscectomy, frequently leading to rapidly progressive degenerative osteoarthritis within the knee joint. The majority of medial meniscal bony avulsions have been reported in younger, skeletally immature patients. Cases of bony avulsions in older patients have sparsely documented.

**Care Presentation:**

In this case report, we highlight the case of a 45‐year‐old male with a history of osteoporosis who sustained a twisting injury to the right knee which subsequently resulted in a bony avulsion injury of his medial meniscal root. MRI imaging was acquired, showing injury to the medial meniscus concurrent with an avulsion of the posterior root. A transtibial pullout repair was performed, anchoring the bony avulsion into its insertion site to allow for direct bone‐to‐bone healing.

**Conclusion:**

This case presents a unique mechanism of injury, as most medial meniscal root injuries in older patients involve a tear of the root rather than avulsion of the bone. Additionally, meniscal bony avulsions have been reported to mainly be a consequence of acute traumatic injury. We hypothesize that this patient′s history of osteoporosis contributed to his bony avulsion, especially because of his age and his relatively nontraumatic mechanism of injury.

## 1. Introduction

The menisci of the knee are circumflex fibrocartilaginous structures lining the tibial plateau in the tibiofemoral joint space [1, 2]. The medial meniscus has two principal attachments in the tibia: the posterior aspect of the medial tibial intercondylar eminence, anterior to the posterior cruciate ligament (PCL) and the anterior intercondylar crest of the tibia, anterior to the anterior cruciate ligament (ACL), with relevant stabilizing attachments through the meniscotibial ligaments, capsule, and deep medial collateral ligament. Meniscus injuries are estimated to be at about 60 per 100,000 within the population, with recent increases in diagnoses because of increased sports activity and stronger diagnostic methods [3, 4]. Meniscus injuries have been known to cause degenerative changes in the knee joint, mainly osteoarthritis [4–6]. With the increasing incidence of meniscus tears and their significant impact on patient quality of life, treatment of meniscus injuries has become a topic of major interest.

Medial meniscus posterior root tears are most frequently seen among older, obese, and generally sedentary patients [7]. Within this subgroup of meniscal injuries, total avulsions of the posterior root are the rarest. Patients with medial meniscus root avulsion injuries have been known to present with posterior knee pain, pain at the joint line, swelling, clicking, and knee instability [4, 8]. On magnetic resonance imaging (MRI), a sagittal view of the medial meniscus may show a “ghost sign,” a missing posterior meniscal attachment. On a coronal view the torn meniscus will appear extruded from its normal anatomic location [8].

The literature on medial meniscus root bony avulsions is sparse. The majority of medial meniscal bony avulsions documented have been found to be in young skeletally immature males following an acute traumatic injury to the knee [8, 9]. There is one case reported in the English‐language literature involving a medial meniscal root avulsion in an older patient. However, the patient had a concurrent anterior cruciate ligament bony avulsion following a traumatic twisting injury [9]. In this study we present the diagnosis and treatment of an isolated bony avulsion to the posterior root of the medial meniscus within a skeletally mature patient.

## 2. Case Study

A 45‐year‐old male with a history of idiopathic osteoporosis (shown on bone biopsy) and history of multiple fractures (including spontaneous femoral neck fracture and multiple pathologic fractures of the feet) who was referred to clinic for a suspected meniscus tear following an acute right knee injury. The patient states that he was fixing a vehicle when he twisted his knee and heard a popping sound. The patient had complaints of right knee pain posteromedially and laterally with associated swelling. His ligamentous exam was stable with a negative Lachman, pivot shift, and anterior drawer test. The patient had no varus/valgus instability at zero and 20°. He had a negative Godfrey, negative posterior drawer, negative posterolateral and posteromedial rotary instability testing, negative reverse pivot shift testing, and no increased external dial at 30 or 90°. Joint line exam indicated 2 + mid and 1 + posteromedial joint line tenderness and 1 + posterolateral joint line tenderness. There was a positive McMurray′s to pain.

**Figure 1:**
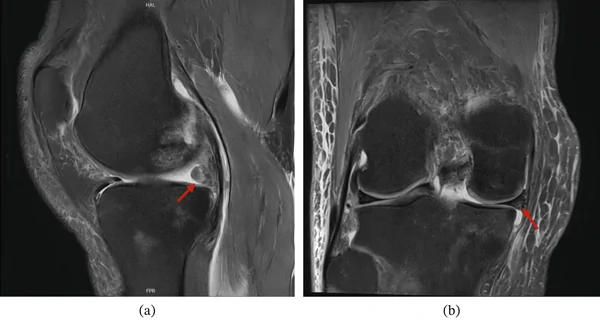
Sagittal and coronal MRI views of the right knee. (a) sagittal view showing the “ghost sign,” discontinuity of the medial meniscus into its posterior attachment. (b) coronal view, highlighting meniscal protrusion (marked by the arrows), suggesting an avulsion of the posterior root.

On MRI, coronal views demonstrate a medial meniscus injury concurrent with a bony avulsion of the posterior horn (Figure 1b). Lateral protrusion of the medial meniscus was also visualized. Sagittal views demonstrated injury to the posterior horn of the medial meniscus (Figure 1a). Because of the patient′s clinical presentation and imaging, a meniscal root injury was suspected with possible bony involvement. The differential diagnosis included potential loose bodies and meniscal ossicles. Arthroscopic meniscal surgery was recommended with possible meniscal debridement or root repair.

Standard anterolateral and anteromedial arthroscopic portals were made with an 11‐blade knife with the knife blade pointed upwards to protect the menisci. A small synovectomy was performed for visualization purposes. In the figure‐of‐four position, the lateral compartment was visualized. The lateral meniscus was probed in the superior and inferior aspects as well as the root attachments. There was a radial split tear in the middle horn of the lateral meniscus measuring approximately 10% of the width. A partial lateral meniscectomy was performed as to give a smooth stable rim. Following the partial lateral meniscectomy, the probe was used to confirm no residual loose flaps or unstable rims. There was no evidence of any lateral compartment chondral pathology.

**Figure 2:**
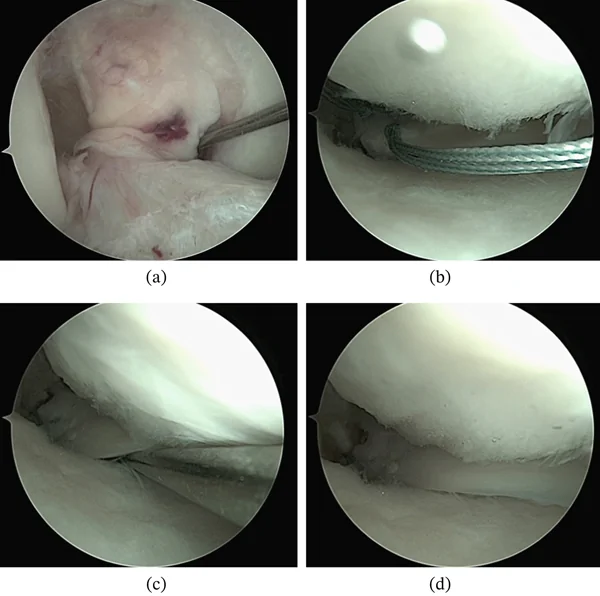
Arthroscopic views of right medial meniscus before and after repair. (a) visualizes the avulsed medial meniscus with bony fragment. (b) represents the meniscal root captured by two luggage tag FiberWire sutures. (c) represents probe test on the medial meniscus after anchoring. (d) represents the meniscus after repair.

Next, in slight flexion with valgus stress placed on the knee, the medial compartment was visualized. On probing, the medial meniscus bony avulsion was visualized (Figure 2a). In order to increase visualization and access to the medial compartment, the medial collateral ligament was pie crusted. Given his young age and acute nature of a medial meniscus avulsion, a repair was warranted. Firstly, the undersurface of the bone was debrided, while making sure that a small sliver of the bony avulsion was preserved to allow for bone‐to‐bone healing. The cartilage at the insertion site was cleaned using an ArthroCare device (Smith & Nephew, Austin, Texas), and subsequently a rasp was used to create a bleeding bony trough. Two luggage tag FiberWire sutures (Arthrex, Naples, Florida) were then passed through the meniscal insertion site just medial to, as well as anterior and posterior to the bony avulsion (Figure 2b).

Then using a low‐profile point‐to‐point guide, a 2.4 drill pin was used to drill a hole and bony tunnel from the anterior aspect of the tibia exiting at the meniscal insertion site just lateral to the medial tubercle into the bony trough. An oversleeve was malleted approximately 1 cm into the tibia and guide pin was removed to allow for a suture wire to be passed and luggage tag sutures to be shuttled through the bone tunnel. Sutures were then fixed to the tibia at 30° of flexion with a 4.75 BioComposite SwiveLock (Arthrex, Naples, Florida). This was done under direct visualization with the scope to ensure appropriate tension of the avulsed root into the previously created trough and direct bone‐to‐bone healing, and the avulsed root was probed after anchoring (Figure 2c). The repair was observed through the scope both before and after the knee was taken through a full range of motion test to ensure adequate repair (Figure 2d). Once the repair was deemed appropriate, the scope was placed in the posterior aspect of the knee. There were no residual loose bodies or any other pathology. All arthroscopic equipment was then removed, and the arthroscopic portals were closed using 3‐0 nylon sutures.

Postoperatively the patient was placed in an ACE wrap from toes to mid‐thigh, with a polar care device and TROM brace locked in extension. Weight bearing was protected with toe touch recommendations for a total of 6 weeks. At 12 days postopoperatively, the brace was unlocked to allow for progression of his flexion to 90° and started on home exercises and formal PT. At 6 weeks postoperatively, he progressed to full weight bearing and unlimited range of motion and placed in a small, hinged knee brace for activities. He reported great improvement from prior to surgery with decreased pain, improved function, and elimination of his mechanical symptoms. He was started on stationary bike at 6 weeks post‐op, elliptical machine at 10 weeks post‐op, straight ahead running program at 14 weeks post‐op, and progression to dynamic activities at 20 weeks post‐op. He was cleared for all activities at 6 months post‐op and has had no complaints or limitations related to his knee.

## 3. Discussion

Avulsion injuries to the medial meniscus are extraordinarily rare, with only 11 reported cases per the present study′s review [9]. In the review by Feucht et al., he found that the majority (58%) of avulsion fractures were in patients who were under the age of 18. This lends credit to the hypothesis that meniscal avulsion fractures in adolescents are more common because of the bone being immature, and hence weaker than the ligamentous attachment [9]. In this case we report a novel injury mechanism of a skeletally mature patient suffering from a standalone medial meniscus posterior horn bony avulsion. We hypothesize that our patient′s avulsion injury was due to his idiopathic osteoporosis, causing his bony architecture to have weakened to the point where the bone was more easily avulsed at the posterior meniscus attachment rather than the meniscus root being torn. Root tears have historically been treated nonoperatively or by a partial or total meniscectomy [10]. However, because of the significant evidence of degenerative osteoarthritis due to increased load bearing in patients with torn menisci, surgical repair/refixation has become heavily favored. This is especially true in patients who are young or who have sufficient healthy cartilage. Currently, there are two main techniques employed to fix medial meniscus root tears: transtibial pullout refixation and suture anchor repair [9–11]. The latter technique has been shown to guard against micromovements of the meniscus after repair, decreasing long‐term abrasion of the suture‐meniscal complex [12, 13]. However, the transtibial pullout technique has been favored by surgeons because of its relative safety, technical simplicity, and low risk that the sutures would loosen over time, like in an anchor repair [11]. This method was used in our case, with the addition of allowing for direct bone‐to‐bone healing rather than only anchoring the torn meniscus on the tibial plateau. Additionally, use of the Biocomposite Swivel‐Lock allowed for secure fixation of the avulsed bone into the trough. This mechanism allowed for both direct bone‐to‐bone healing, as well as a stronger foundation for the suture fixation.

In this paper we present a skeletally mature patient that suffered from a medial meniscus bony avulsion and describe a transtibial pull out technique that allows for direct bone‐to‐bone healing. To our knowledge this is one of the only papers that describes a case of an isolated medial meniscal bony avulsion in a skeletally mature patient. The technique employed in this case allows for direct healing of the avulsed bone, greatly reducing the chances of developing osteoarthritis due to the root tear.

## Funding

No funding was received for this manuscript.

## Conflicts of Interest

Anikar Chhabra, MD: consultant for Smith & Nephew and Arthrex; Speakers Bureau for Arthrex; Board Member for AZ Sports and Tourism Authority. The remaining authors (Mr. Ahmad R. Alhankawi, BA; M. Lane Moore, MD, MBA; Mr. Don L. Dulle III, PA‐C; Matthew B. Anastasi, MD) declare that they have no conflicts of interest regarding the publication of this article. The authors′ consulting and speaker relationships with Smith & Nephew and Arthrex did not influence the selection of devices, clinical management, or the preparation of this case report. No funding, equipment, editorial assistance, or other support was provided by these companies, and the authors′ institution has no relevant agreements with them. All relevant conflicts of interest have been fully disclosed in accordance with the journal′s policy.

## Data Availability

The data that support the findings of this study are available on request from the corresponding author. The data are not publicly available due to privacy or ethical restrictions.
